# Functional claudication distance: a reliable and valid measurement to assess functional limitation in patients with intermittent claudication

**DOI:** 10.1186/1471-2261-9-9

**Published:** 2009-03-02

**Authors:** Lotte M Kruidenier, Saskia PA Nicolaï, Edith M Willigendael, Rob A de Bie, Martin H Prins, Joep AW Teijink

**Affiliations:** 1 Department of Surgery, Atrium medical centre Parkstad, PO Box 4446, 6401 CX Heerlen, the Netherlands; 2 Department of Epidemiology and Caphri research School, Maastricht University, Maastricht, the Netherlands

## Abstract

**Background:**

Disease severity and functional impairment in patients with intermittent claudication is usually quantified by the measurement of pain-free walking distance (intermittent claudication distance, ICD) and maximal walking distance (absolute claudication distance, ACD). However, the distance at which a patient would prefer to stop because of claudication pain seems a definition that is more correspondent with the actual daily life walking distance. We conducted a study in which the distance a patient prefers to stop was defined as the functional claudication distance (FCD), and estimated the reliability and validity of this measurement.

**Methods:**

In this clinical validity study we included patients with intermittent claudication, following a supervised exercise therapy program. The first study part consisted of two standardised treadmill tests. During each test ICD, FCD and ACD were determined. Primary endpoint was the reliability as represented by the calculated intra-class correlation coefficients. In the second study part patients performed a standardised treadmill test and filled out the Rand-36 questionnaire. Spearman's rho was calculated to assess validity.

**Results:**

The intra-class correlation coefficients of ICD, FCD and ACD were 0.940, 0.959, and 0.975 respectively. FCD correlated significantly with five out of nine domains, namely physical function (rho = 0.571), physical role (rho = 0.532), vitality (rho = 0.416), pain (rho = 0.416) and health change (rho = 0.414).

**Conclusion:**

FCD is a reliable and valid measurement for determining functional capacity in trained patients with intermittent claudication. Furthermore it seems that FCD better reflects the actual functional impairment. In future studies, FCD could be used alongside ICD and ACD.

## Background

Intermittent claudication is a symptom of peripheral arterial disease (PAD), and is described as muscle pain in the lower extremities that is produced by exercise and relieved in rest. Patients with intermittent claudication have limited exercise and walking capacity, which reduces their functional capacity[1].

Treadmill testing is a common way to quantify the grade of functional impairment. The Royal Dutch Society for Physiotherapists recommends the administration of treadmill tests to all patients with intermittent claudication, both to objectively document the degree of functional impairment, and to evaluate therapy effect[2].

In general, two distances are measured during treadmill testing of patients with intermittent claudication. First is the distance walked at the onset of claudication pain, also known as the initial claudication distance (ICD), or pain-free walking distance. The second measurement is the distance at which claudication pain becomes so severe that the patient is forced to stop, also known as the absolute claudication distance (ACD), or maximal walking distance [2-6]. In the literature, both ICD and ACD are used to classify the degree of functional impairment. Both distances have been shown to be reliable measurements with good reproducibility. ICD appears to be less reliable in comparison with ACD [7-13].

In patients with intermittent claudication both ICD and ACD correlate with different quality of life domains of the EuroQol[14], the Short-form-36[15,16], and several disease specific questionnaires [17-19]. However, the definition of both ICD and ACD is not correspondent with distances a patient would walk in daily life. Although most patients will continue to walk after appearance of the first signs of pain, few will walk until their maximum pain threshold is reached during the course of daily activities.

For this reason, the distance at which a patient prefers to stop because of claudication pain may be a better instrument by which to measure the functional impairment of patients with intermittent claudication. Bendermacher et al[20] first used "the distance at which a patient prefers to stop because of claudication pain". We define this distance as the functional claudication distance (FCD).

We conducted this study, since the reliability and validity of FCD have never been tested. Furthermore we want to compare reliability and validity of FCD with both ICD and ACD to determine the value of FCD for testing functional impairment in patients with intermittent claudication.

## Methods

### Patients

Patients with intermittent claudication, following a supervised exercise program, were recruited from private physiotherapy practices in the Southern part of the Netherlands. Inclusion criteria were intermittent claudication with an ACD of < 1600 meters on a standard treadmill test. Patients had to have followed at least 3 months of community based supervised exercise therapy according to the guidelines of the Royal Dutch Society of Physiotherapy to rule out therapy effect between the 2 study measurements. Exclusion criteria were the inability to walk on the standard treadmill protocol, serious cardiopulmonary comorbidity (NYHA 3 and 4)[21] and reasons for discontinuing the treadmill test other than intermittent claudication. The study was approved by the local research ethics committee from the Atrium medical centre Heerlen, and all patients provided informed consent.

### Study protocol

The study consisted of two parts. In the first part, 57 patients were included who performed two standardised treadmill tests within three weeks. Patients rested for 10 minutes before each test to ensure that no claudication pain was present at the start. Handrail support was not allowed. In case of unbalance, the researcher gave the patient his hand to hold on to until balance was regained. During the treadmill tests, patients were blinded for the distance/time walked by covering the display of the treadmill. The data from the first part were used to determine reliability of ICD, FCD, and ACD.

In the second part, 25 patients were included who all performed a standardised treadmill test and filled out a Rand-36 questionnaire to determine quality of life. The Rand-36 is a general quality of life questionnaire and determines quality of life in 9 domains of functioning[22]. Data from the second part were used to determine validity of FCD, compared to ICD and ACD.

### Treadmill testing

A progressive treadmill test was used according to Gardner et al[23]. with a constant speed of 3.2 km/h and an increase in inclination of 2% every two minutes, beginning with 0% inclination. The inclination and testing duration were maximised to 10% and 30 minutes (1600 metres), respectively. Patients participating in the first part of the study performed two treadmill tests. Patients participating in the second part performed only one treadmill test. During treadmill testing all patients were supervised by one of two independent researchers. At each test all walking distances (ICD, FCD, and ACD) were measured. Patients indicated the onset of claudication pain, the point of preferring to stop, and the point that maximum walking distance was reached.

### Analysis

Nominal and interval variables are presented as frequency (%) and mean ± standard deviation respectively, unless otherwise indicated. Differences in baseline characteristics between the two groups were assessed by a Chi-square test for nominal variables and a Paired Student's T-test for interval variables.

To determine the reliability of ICD, FCD, and ACD, an intra-class correlation coefficient (ICC) for absolute agreement was calculated, according to a two-way mixed effects model with random effects for subjects and a fixed effect for time[24]. Bland-Altman plots were used to visualize the repeated measurements[25]. Regression analysis was applied to assess whether the difference between the two measurements is dependent on the mean walking distance to determine if a log transformation of the Bland-Altman plots is necessary. The extent of variability between repeated measurements was assessed by the coefficient of variation for ICD, FCD and ACD separately.

In the validity study scatter plots were used to examine the linearity of the correlation and to detect possible outliers. Walking distance was plotted against the value of the different domains of the Rand-36 questionnaire for each patient individually. Outliers, appearing as points far away from the overall pattern were excluded. Validity was assessed using the Spearman's rho to calculate the degree of correlation between ICD, FCD, and ACD and the different domains of the Rand-36 questionnaire. Data were analysed using SPSS 12.0.

## Results

In total eighty-two patients were included in this study, of whom 57 in the reliability part and twenty-five in the validity part. The patient characteristics are shown in Table 1, and as can be seen, no significant differences were present between the two patient groups.

**Table 1:** Clinical characteristics of included patients

**Characteristic**	**Total Population** **N = 82**	**Patients for reliability analysis** **N = 57**	**Patients for validity analysis** **N = 25**	**P-value**
Male	49 (59.8%)	36 (63.2%)	13 (52.0%)	0.343
Age (years)	67 ± 10	68 ± 9	65 ± 12	0.213
ABI	0.69 ± 0.19	0.71 ± 0.20	0.66 ± 0.17	0.338
Weight (kg)	76 ± 14	76 ± 14	75 ± 15	0.797
Risk factors				
Hypertension	60 (78.9%)	39 (76.5%)	21 (84.0%)	0.449
Diabetes Mellitus	20 (26.3%)	10 (19.6%)	10 (40.0%)	0.058
Hypercholesterolemia	43 (56.6%)	25 (49.0%)	18 (72.0%)	0.058
Smoking behaviour				0.669*
Current smoking	34 (44.7%)	22 (43.1%)	12 (48%)	
Former smoking	35 (46.1%)	24 (47.1%)	11 (44.0%)	
Never smoked	7 (9.2%)	5 (9.8%)	2 (8.0%)	

Kg: Kilograms, ABI: ankle brachial index.

* Calculated with Kendall's-tau test.

For one patient participating in the reliability study no FCD was measured, for reasons unknown, resulting in 56 patients available for the reliability analysis of FCD.

The mean walking distances (ICD, FCD, and ACD) are shown in Table 2. For every patient the FCD laid in between ICD and ACD. The mean difference between FCD and ACD was 104 and 106 metres for the first and second treadmill test respectively.

**Table 2:** Mean walking distances and reliability measurements

	**Measurement one (metres)**	**Measurement two (metres)**	**ICC Value (95% CI)**	**Coefficient of variation (%)**
ICD	271.6 ± 174.9	273.7 ± 162.2	0.940 (0.899 – 0.964)	21.7%
FCD	531.2 ± 357.3	541.2 ± 339.6	0.959 (0.931 – 0.976)	18.8%
ACD	635.4 ± 376.0	642.6 ± 368.8	0.975 (0.957 – 0.985)	13.2%

SD: standard deviation, ICC: intra-class correlation coefficient, CI: confidence interval, ICD: initial claudication distance, FCD: functional claudication distance, ACD: absolute claudication distance

**Figure 1:**
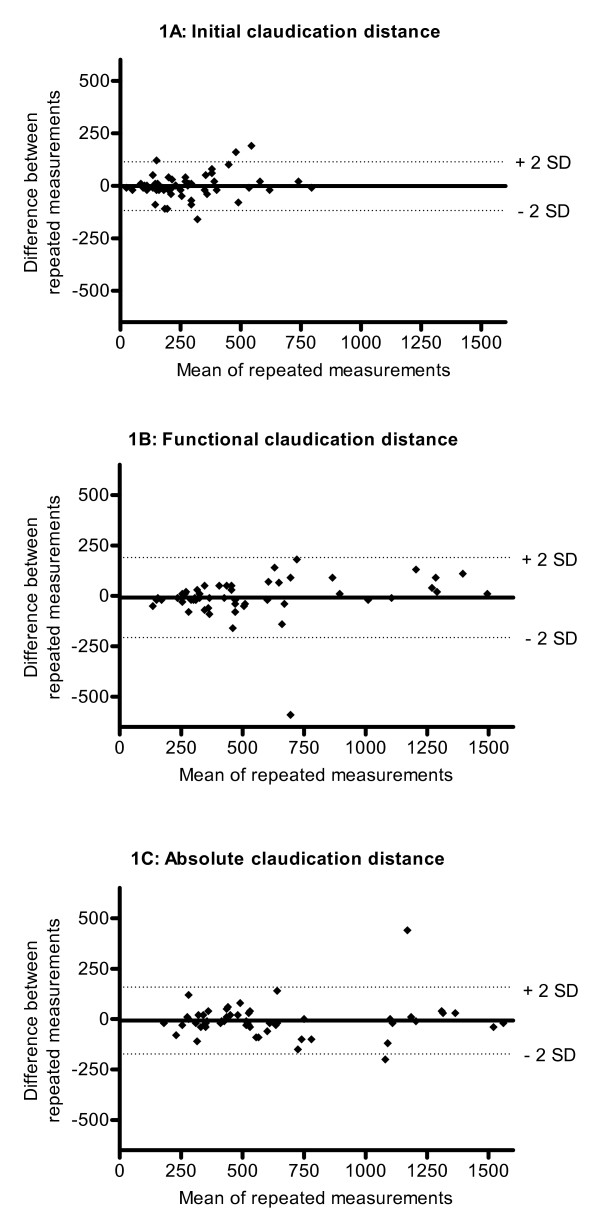
**Walking distances, represented by Bland-Altman plots**. For ICD, FCD and ACD respectively, the mean of the two measurements is plotted against the difference between the two measurements.

Figure 1A, B, and 1C show Bland-Altman plots of ICD, FCD and ACD, respectively. The mean value of 2 measurements is plotted against the difference of measurement 1 minus measurement 2. Regression analysis did not show that systematic differences between repeated measurements were dependent on mean walking distance, indicating that a log transformation of the Bland-Altman plots was unnecessary[25]. Differences between repeated measurements as presented in the Bland-Altman plots are equally divided above and below zero difference. This shows that no learning/therapy effect occurred between the two measurements.

The reliability measurements presented in Table 2 show that the ICC of ACD (0.975, 95% CI 0.957 – 0.985) was significantly better than the ICC of ICD (0.940, 95% CI 0.899 – 0.964). The ICC of FCD was set in between these two, with a value of 0.959 (95% CI 0.931 – 0.976), not significantly different from ICD or ACD. The coefficients of variation showed corresponding results with values of 21.7%, 18.1% and 13.2% for the ICD, FCD and ACD, respectively.

Based on the scatter plots two patients were identified as outliers and excluded from the analysis, leaving 23 patients for validity analysis. The mean scores of the Rand-36 questionnaire and the correlations of the different walking distances with quality of life are shown in table 3. The ICD correlated significantly with the physical function (rho = 0.473, p = 0.022) and general health (rho = 0.518, p = 0.011) domain of the Rand-36 questionnaire. FCD correlated significantly with five out of nine domains, namely physical function (rho = 0.571, p = 0.004)), physical role (rho = 0.532, p = 0.009), vitality (rho = 0.416, p = 0.048), pain (rho = 0.416, p = 0.037) and health change (rho = 0.414, p = 0.050). ACD correlated with physical function (rho = 0.496, p = 0.016), physical role (rho = 0.519, p = 0.011) and health change (rho = 0.446, p = 0.033).

**Table 3:** Rand-36 scores and Spearman's correlations with walking distances

**Domain**	**Rand-36 score**	**ICD**		**FCD**		**ACD**	
	**Median (IQR)**	**Correlation**	**P value**	**Correlation**	**P value**	**Correlation**	**P value**
Physical function	55.6 (50.0 – 72.2)	0.473*	0.022	0.571**	0.004	0.496*	0.016
Social function	87.5 (75.0 – 100.0)	-0.046	0.836	0.001	0.998	-0.065	0.768
Physical role	75.0 (0.0 – 100.0)	0.407	0.054	0.532**	0.009	0.519*	0.011
Emotional role	100.0 (100.0 – 100.0)	0.068	0.758	0.157	0.476	0.121	0.584
Mental health	84.0 (64.0 – 92.0)	0.014	0.948	0.132	0.549	0.092	0.676
Vitality	60.0 (55.0 – 70.0)	0.152	0.488	0.416*	0.048	0.366	0.086
Pain	67.3 (55.1 – 69.4)	0.338	0.114	0.437*	0.037	0.352	0.099
General health	50.0 (40.0 – 60.0)	0.518*	0.011	0.392	0.065	0.371	0.081
Health change	50 (25.0 – 75.0)	0.382	0.072	0.414*	0.050	0.446*	0.033

IQR: inter quartile range, ICD: initial claudication distance, FCD: functional claudication distance, ACD: absolute claudication distance.

* Correlation is significant at the 0.05 level,

** Correlation is significant at the 0.01 level.

## Discussion

FCD, defined as the distance when the patient prefers to stop due to claudication, is a reliable and valid measurement to determine functional impairment in patients with intermittent claudication.

The ICC of FCD was 0.959 and in between of the ICC of ICD and ACD, with ACD showing the most reproducible measurements. The coefficients of variation showed corresponding results. ACD showed the least variation, followed by FCD and ICD, respectively.

FCD correlated significantly with the physical function, physical role, vitality, pain and health change domain of the Rand-36 questionnaire. ICD correlated significantly with the physical function and general health domain. Significant correlations of ACD were found with physical function, physical role, and health change. These results indicate that FCD corresponds best with general quality of life as FCD correlated with five of nine domains compared to two and three domains for ICD and ACD respectively.

In our study, ACD is the most reliable measurement during a treadmill test. This conforms to results found in literature for several treadmill protocols [7-13]. Three studies from Gardner et al[10,23] and Labs et al[11] assessed the reliability of the treadmill protocol used in this study. The ICC of ICD and ACD in these studies ranges from 0.82 to 0.89 and from 0.93 to 0.96, respectively. The coefficients of variation in these studies range from 11.0% to 15.5% for ACD, and from 15.8% to 28.6% for ICD. These findings are in line with coefficients of variation found in our study, and indicate that ICD and ACD are both reliable measurements. However, the ICC found in our study tends to be better than those previously described in the literature. One possible explanation for this difference could be that our test population consisted of patients familiar with treadmill testing. Prior to this study, all patients received at least 3 months of community based supervised exercise therapy, consisting mainly of treadmill walking. This may have influenced the stability of the outcomes of the treadmill tests. It seems plausible that reliability between two measurements increases with treadmill training of the patients, as compared to untrained patients.

In our study FCD correlates best with quality of life, followed by ACD and ICD. In literature several studies determined correlations between QOL and walking distances. A recent study in 48 patients from Myers et al[16] showed a significant correlation of ICD with both pain and social function whereas ACD correlated with physical function and vitality measured by the short-form-36. Izquierdo-Porrera et al[15] determined Pearson's correlation coefficients between ACD and the different domains of the short-form-36. In this study ACD correlated significantly with physical function (r = 0.43), physical role (r = 0.33), and mental health (r = 0.27). Furthermore, ICD and ACD correlate with different domains of the PAVK-86[19], the CLAUS questionnaire[17], and the VascuQol[17].

Limitation of the study is that we included patients familiar with treadmill walking, what could have influenced the reliability results. FCD results should therefore be measured in other patient populations and until then treated with caution in these populations. However, corresponding coefficients of variation for ICD and ACD from the literature (untrained patients) compared to our study (trained patients) may indicate that the results from this study for FCD can be projected to patients unfamiliar with treadmill walking.

A further limitation is the limited number of patients included in the validity study (n = 23 for the analysis). A study including more patients to confirm our results is desirable.

The definition of FCD, the distance at which a patient prefers to stop walking, assumes a better reflection of the functional capacity of patients than ICD or ACD. In practice, most patients do not stop walking at the first indication of claudication pain, neither do they walk until they reach their maximal pain threshold. In our study, comparison of FCD with the Rand-36, for the purpose of establishing the clinical relevance shows that FCD correlates better with quality of life than both ICD and ACD. Therefore we think that FCD is a more important outcome measurement from a patient's perspective. Furthermore, from a research perspective, FCD is a reliable and valid instrument that can be used in clinical trials. In the future it is conceivable that training programs using a global positioning system will be developed using a software program calculating walking distances and recuperation time[26] Especially in such a training environment it is more likely that the average claudicant will stop when preferring so than until reaching a maximal pain threshold.

## Conclusion

The functional claudication distance is a reliable measurement for determining functional capacity in trained patients with intermittent claudication. Furthermore it seems that FCD better reflects the actual functional impairment. In future studies, FCD could be used in conjunction with ICD and ACD.

## Competing interests

The authors declare that they have no competing interests.

## Authors' contributions

LK contributed to the conception and design of the study, analysed and interpreted the data and wrote the manuscript. SN contributed to the conception and design of the study, collected data and revised the article critically. EW contributed to the data collection and revised the article critically. RdB contributed to the data collection and revised the article critically. MP contributed to the conception and design, checked the analysis and interpretation and revised the article critically. JT contributed to conception and design, revised the article critically and had overall responsibility over the study.

All authors read and approved the final manuscript.

## Pre-publication history

The pre-publication history for this paper can be accessed here:


